# On mycohuman performances: fungi in current artistic research

**DOI:** 10.1186/s40694-019-0085-6

**Published:** 2019-12-04

**Authors:** Regine Rapp

**Affiliations:** 1 Art Laboratory Berlin, Berlin, Germany; 2 Institute for Biotechnology, Technische Universität Berlin, Berlin, Germany

## Abstract

This review reflects several artists and their artistic research in the field of hybrid art, bio art, or science art, working with Fungi as both subject matter and medium. The work of Saša Spačal, Tarsh Bates and Theresa Schubert is not representational in the manner of traditional fine art, but works rather through performative, multidisciplinary and research-based strategies to produce artwork through fungal material as such. My research results are based on the series “Nonhuman subjectivities” and “Nonhuman agents” that Christian de Lutz and I conceived and realized at Art Laboratory Berlin (2016–18) in various formats—exhibitions, workshops, lectures and a conference. The work of Saša Spačal and her colleagues involves creating interactive situations of symbiosis between the fungal and the human. An example of this is *Myconnect*, in which a biofeedback loop is related between the human participant and Oyster mushroom mycelia through a special encounter, which is mediated by non-linguistic forms of awareness and exchange—sonic, electronic and metabolic. Tarsh Bates’ work with *Candida albicans* and *Candida parapsilosis* refers to a complex and intimate relation between the human and yeasts that form part of the human microbiome. Bates considers the relationship between humans and yeast as “CandidaHomo Ecologies” and sees both partners as equals. She explores this relationship through her work *The Surface dynamics of adhesion,* examines it from historical and metabolic levels through an installation that includes the live yeast growing on agar mixed with the artist’s own blood. Theresa Schubert’s installations and site-specific interventions treat living organisms, especially Fungi, as collaborators and co-creators. Her work *Growing Geometries*—*Tattooing Mushrooms* follows the morphological development of fungal fruiting bodies through the intervention of a tattoo. Her performative forest walks, especially the *Forestal Psyche* and also new actions for the “Mind the Fungi” project, engage the public in an intimate and multi sensory encounter with Fungi and their surrounding environment.

## Background

In the last years fungi have received more and more international attention in artistic research—specifically in the art domains known as *Bio Art*, *Science Art* or *Post Media Art*, under the general domain of *Hybrid Arts*. Fungi, especially mycelia, have become vital agents in some outstanding artistic experiments, installation projects, and ongoing art interventions.

Especially noteworthy are the different unconventional practices of these artists who, in their process-oriented artistic projects, often turn to new formats and themes in the form of a *multidisciplinary* practice. The selected examples show how the traditional laboratory and exhibition space are functionally, performatively and interactively questioned, expanded or even infiltrated. The respective artists usually not only work collaboratively with scientists, they also work in science labs themselves.

Therefore, let us consider this a new artistic paradigm: One in which artistic practices that have a direct interest in *organic matter* as such—in our case *fungal material*. The artists discussed deal with biomaterials in open, accessible formats, without subsequently transforming them into a traditional artistic format (such as paintings or pictures). Representation is consciously undermined in favour of an immediate artistic exploration of *matter*. It is significant that in this new art movement, one increasingly encounters the open and fleeting format of performance and collaborative forms of work [1].

In this article I want to introduce three outstanding artists working continually with fungi in their artistic practice—Saša Spačal, Tarsh Bates and Theresa Schubert. All were closely connected to the series “Nonhuman Subjectivities” and “Nonhuman Agents”, Christian de Lutz and I had conceived and realized recently at Art Laboratory Berlin.^1^ Fungal matter is considered here in various ways: as co-habitant, as microorganism within the human body, or object of geometrical experiment.

## Three outstanding artists working with fungi in the context of hybrid arts

### Oyster mycelium performing with humans

*“My artistic practice in some aspects takes form of mycohuman relationship, which is based on the entanglement of mycorrhizal extensions that transgress both fungi and humans as species, thus forming an extensive rhizomatic network so vast that it encompasses several planes of existence: material, immaterial, organic, technological, social and planetary. On this planet of interspecies negotiation, symbiotic bonds are implemented with mycorrhizal technology that enables me to explore my habitat with fungi as my guides and teachers.”* [2].

In a posthuman or post-anthropocentric worldview, we still retain our subjective point of view, but find it de-centred and appropriated by new forms of community. We can no longer consider ourselves to be individual, but a collective of human, fungal, bacterial and viral agents that make us who we are. From that point we move outward into a world, in which we are but part of a stream of interaction and becoming, in tandem with myriad others. Specifically these conditions form the setting for the performative aesthetics and ethics of Saša Spačal’s artwork. Her projects offer intriguing biotechnological strategies for immersion and inter-connectedness. Without ever losing the artistic value of aesthetic experience, Spačal makes use of scientific knowledge and lab practice to setup special encounters that are mediated by non-linguistic forms of awareness and exchange—sonic, electronic and metabolic. The results offer us a new repertoire for re-engaging the world.

**Fig. 1:**
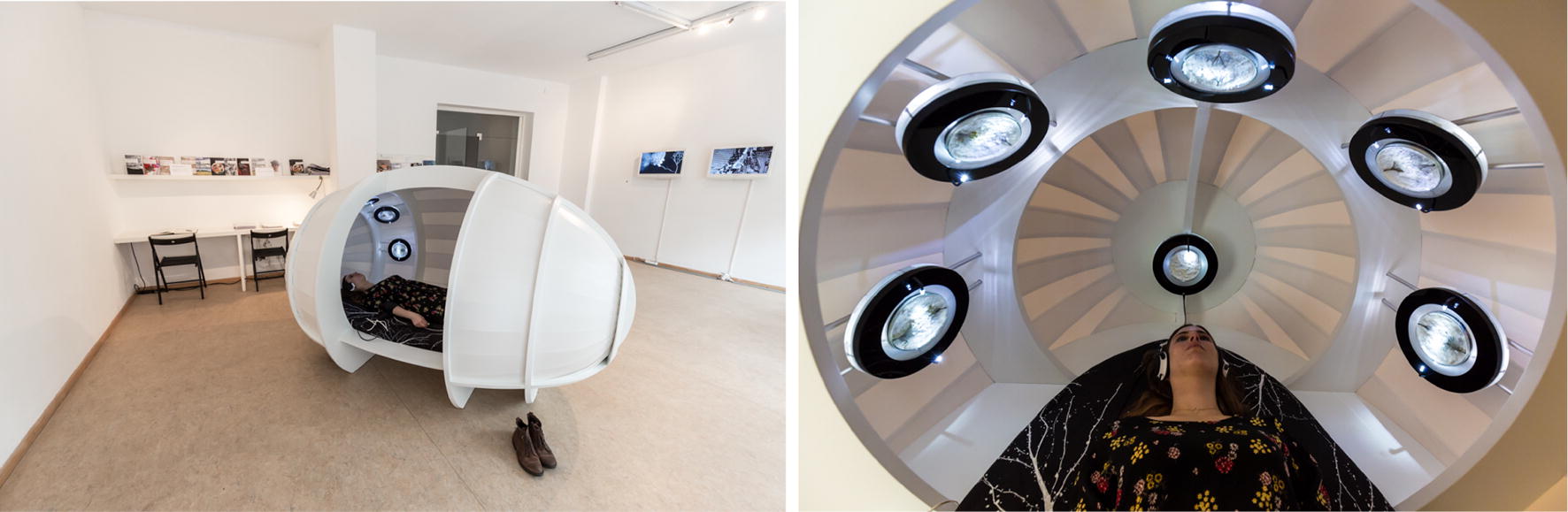
Saša Spačal, Mirjan Švagelj and Anil Podgornik, *Myconnect*, 2013, installation, installation view at Art Laboratory Berlin in 2017. Inside the installation—headphones with sound, technical equipment and 5 petri dishes with oyster mycelium. Photos: Tim Deussen

In the interactive audio–visual installation *Myconnect* (2013) (Fig. 1), the artist together with her colleagues Mirjan Švagelj (microbiologist) and Anil Podgornik (programmer, designer) succeed in experiencing the physical dimension of being connected with the surrounding environment (“Umwelt”) and experiencing the phenomenon of what D. Haraway calls ‘companion-species’. The work is an experiential space in the literal sense: Visitors are invited to individually engage in a biofeedback loop for about 10 min in an almost closed wooden capsule with fungi; Spačal uses Oyster mushrooms (genus *Pleurotus*) or Shiitake (*Lentinula edodes*). This involves connecting a person’s nervous system to a fungal mycelium in a biofeedback loop. Upon entering the capacitive capsule, a person is equipped with a heartbeat sensor, headphones and vibration motors placed on different parts of the body. The human heartbeat sets the system in motion. The signal travels through the Oyster or Shiitake mycelium (in petri dishes), where it is modulated in real time.^2^ The modulated signal is transmitted back to the human body via sound, light and tactile sensory impulses. The overwhelming stimuli that affect the nervous system cause a change in the heartbeat. A new loop begins and the circle is closed. A symbiosis of signals begins.

The installation *Myconnect* is a symbiotic connector of various kinds that questions the anthropocentric division between nature and humans. With its circuit of signals and impulses, generated and translated by biological and technological organisms, *Myconnect* offers an immersive experience of symbiotic interactions. This experience makes the technological distinction between nature and human appear as an arbitrary definition serving particular biopolitical interests of human society.

The artist–biologist–designer collective has decided to work with mushrooms, as it is one of the dominant life forms in the world: “Fungal mycelium seemed to be a perfect organism to form an interspecies connector for multiple reasons”, remarks Spačal about the work *Myconnect*. Referring to the network like structure of this organisms she points out: “Mycelium as the vegetative part of fungus consists of a mass of branching, thread-like hyphae.”^3^

For Spačal fungi have an ability to help her understand space and time scales, and connections between these scales help her to relearn how to relate to nonhuman agents, while her body is immersed into the interconnected planetary system, observing and feeling the fresh pulse of intra-action [2]. The artist makes a direct reference to the philosophical concept of Karen Barad who proposes “agential intra-activity” to overcome representationalism: “[…] the universe is agential intra-activity in its becoming. The primary ontological units are not ‘things’ but phenomena—dynamic topological reconfigurings/entanglements/relationalities/(re)articulations. And the primary semantic units are not ‘words’ but *material*-*discursive practices* through which boundaries are constituted. This dynamism *is* agency. Agency is not an attribute but the ongoing reconfigurings of the world.” [3]

Remarkable is the intense reception of the visitors each of whom engaged with the mycelium in the capsule for 10 min: ranging from peaceful meditative, restful states (in two cases, people had actually fallen asleep) to panic and disturbance. Most commonly, the visitors mentioned “heartbeats” relating to the sound, “womb” or “egg” relating to the form and “meditative” or “secure” in relation to the vibrating points placed on their joints. Almost all visitors kept their eyes closed throughout the experience. Many visitors would remain inside after the experience was over, to collect their bearings, to look at the mycelium, listening to the sound.^4^

A central point in the artistic practice of Saša Spačal is the phenomenon of connection: “In my view, all of this [sic] systems—biological, technological, social, artistic, etc.—are closely interconnected and co-dependently intertwined in the connections continuum. Everything emerges and resides in the connections continuum on different planes however connected to everything else in the network. Our artworks are like organisms that are part of technological ecosystems” [4].

### CandidaHomo Ecologies

Australian artist and life scientist Tarsh Bates artistically explores what it means to be a human being, always bearing in mind that the human body is made up of over one trillion cells, of which only about half are human. Her leitmotifical material, which she explores artistically and scientifically for more than 10 years, is the yeast *Candida albicans* [5].

*Candida albicans* belongs to the yeasts (phylum Ascomycota) and is a species of several hundreds that are found in and on the human body. At least half of all people carry *Candida albicans*, usually without being aware of it. The human body offers the yeast different ecological niches—mouth, intestines, skin. Bates considers the relationship between humans and yeast as “CandidaHomo Ecologies” and sees both partners as equals: “We (Candida and Homo) are in relentless re-orientation, responding to changes in pH, temperature, moisture and nutrients, tentatively traversing the affordances of each other’s bodies” [6].

**Fig. 2:**
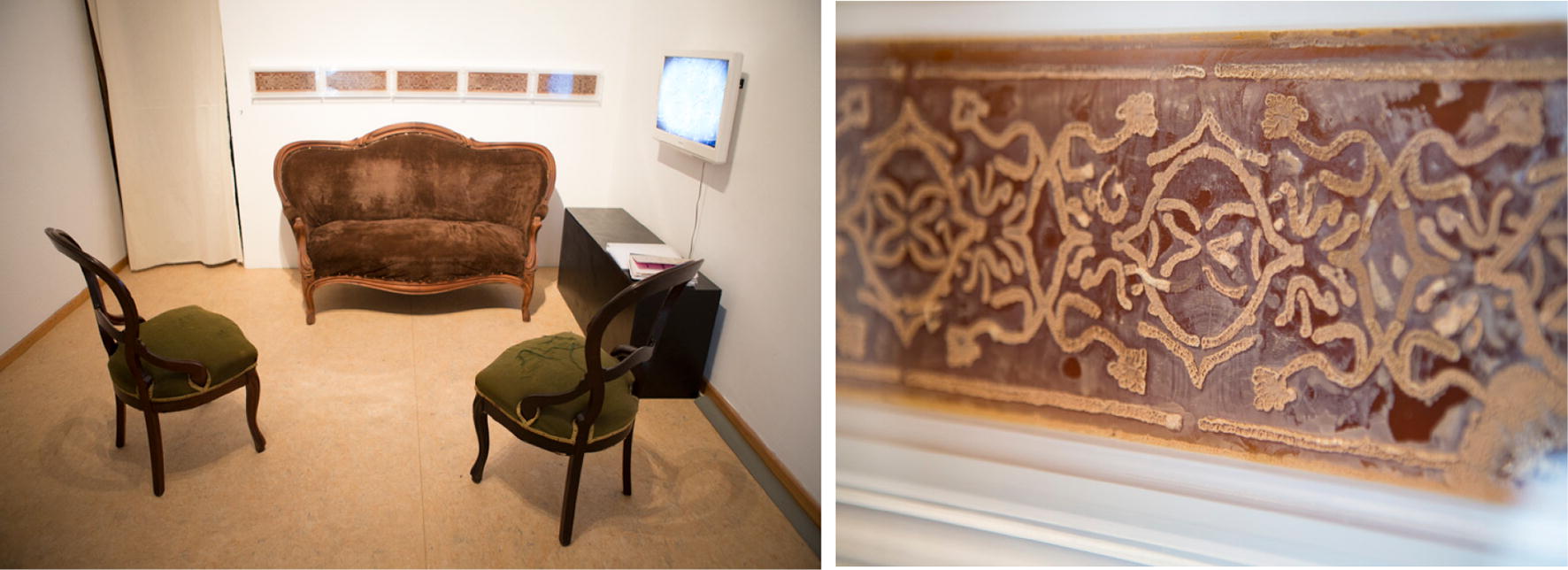
Tarsh Bates: *Surface dynamics of adhesion* (detail), 2016, exhibition view at Art Laboratory Berlin, spring 2016, Photos: Tim Deussen

In 2016 Bates developed her art project *Surface dynamics of adhesion* (Fig. 2) that was shown in the exhibition “The Other Selves. On the Phenomenon of the Microbiome” at Art Laboratory Berlin in spring 2016 [7]. The work puts *Candida albicans* directly in the centre of attention—literally and materially with living candida. The work refers to the cultural, social, psychological and not least microbiological aspects of *Candida albicans*.^5^

**Fig. 3:**
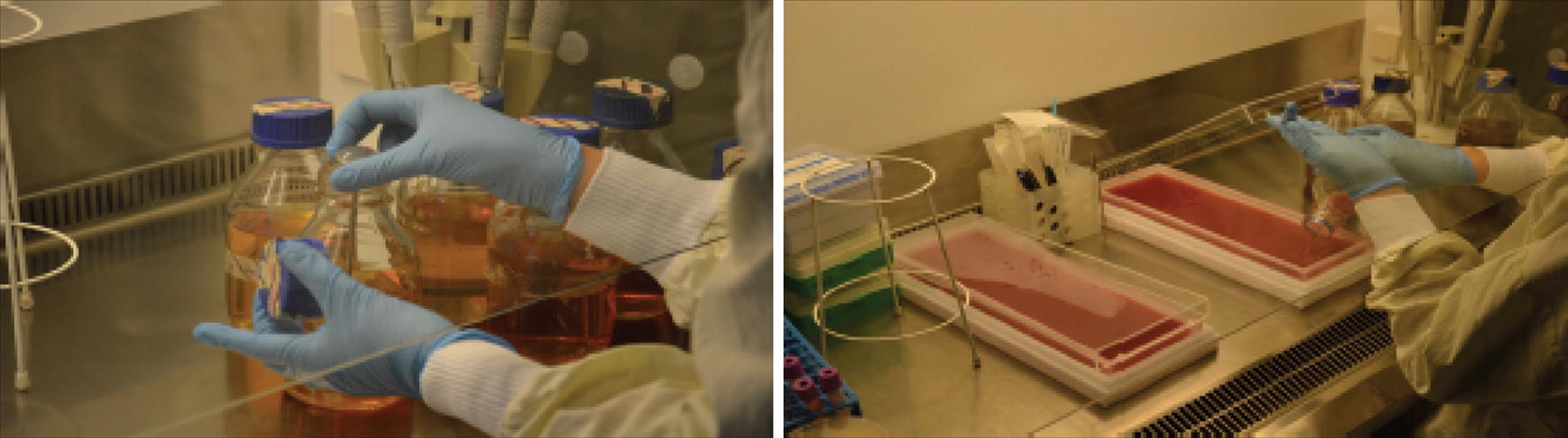
Tarsh Bates at the Deutsche Herzzentrum, Charité Berlin, while preparing the petri dishes with *Candida parapsylosis* on blood agar

In many ways, the installation has a potential for subversion. At first glance, the installation seems to be subject to a clear representationalism—the narrative of a bourgeois interior. A sofa and two Victorian-era chairs are arranged in the room into a suite, with a patterned wallpaper on the wall behind it. Only at second glance, and herein lies the power of this artistic work, one becomes aware of the biomaterials of the ‘wallpaper decoration’. Only by a closer look and orientation in the installation room, it becomes clear that the alleged wallpaper is actually a living organism. I call it a biological trompe-l’œil. The moment of awareness of the delusion takes place in the exhibition room itself—most of the time the visitors are (already) sitting on the sofa, reading background information about the artist, encountering *Candida albicans* and finally learning more about the installation they are already in. As soon as the viewer discovers the living organism in the large rectangular Petri dishes (Fig. 3), the representationalism begins to waver. The playful decor of the bourgeois ambience deliberately misleads the viewer, creating a great moment of becoming aware of the living organism, the yeast. And while the visitors read on the sofa, the organism of which this work deals artistically and scientifically sits literally ‘at their back’! [7]

While in Australia, Bates could present the living *Candida albicans* (level 2 biological protection) in public exhibitions.^6^ For the Berlin project she has decided to use *Candida parapsylosis*, an organism, that was still back in 2016 level 1.^7^ This has to do with regulations that are far more restrictive in Germany and Europe than in Australia: despite her professional sealing of the organism within two layers, it is not permitted in Germany to present level 2 substances (such as *Candida albicans*) in public space outside a scientific laboratory [8].

The red-brown frieze on the wall, mounted at waist height behind the Victorian double seater, resembles a model of flocked wallpaper. The living *Candida parapsylosis*, which is applied in five acrylic plates on agar with the blood of the artist, grows in a pattern very similar to the first drawings of its relative *Candida albicans* by the biologist Charles Philippe Robin from 1853. The formal aesthetic combination of microbiological knowledge and social–historical decoration is fascinating. Especially in the Victorian era, the awareness of hygiene increased rapidly. Thus, Bates marks a historical connection in the mid-19th century in several respects.

Her artistic practice internalizes a form of human–nonhuman collaboration. From the blood of the artist a medium has emerged (in the biotechnological and aesthetic sense), on which the Candida can grow. Bates offers her blood to the yeast to thrive. Bates was able to prepare this complex work in the laboratory of the German Heart Center in the Berlin Charité in Berlin-Steglitz.

This multi-layered work of art can be directly linked to Bard’s term “intra-action”: it is about the relationship between humans and Candida, about the *intra*-*action* of the yeast fungus in and on the human body, also in the exhibition space. In the sense of Barad, matter unfolds in a performative way and undermines the conventional form of representationalism. The hidden message of this installation, the hidden biomaterial, also reflects the many taboos related to Candida in our human society, as an invisible potential for thrush and other infections. This is a remarkable moment in Bates’ work about the subtle (aesthetic) power of biopolitics.

### Co-performing with fungi

While Spačal follows an approach of interconnectedness of human and mycelium and Bates stresses the collaborative cohabitation between human body and *Candida*—there is another outstanding artist who also sympathises with a holistic approach and values the idea of collaborative working with living entities—be it with mushrooms, slime moulds or lichen: Theresa Schubert. The Berlin-based artist researches unconventional visions of nature, technology and the self. She studied media art at the Bauhaus-University, Weimar. In her installations or site-specific interventions she often works with living organisms, who she considers equally as co-workers and collaborators. Schubert critically reflects the world around her through the context of Anthropocene and therefore often refreshingly deconstructs anthropocentric viewpoints. She is also the co-editor of the important publication “Experiencing the unconventional” (2015), that reflects new international art science research with numerous international contributions in electronic, digital and bio art. “*Experiencing the Unconventional. Science in Art’ presents art projects that resulted from unconventional explorations, curious experiments and their creative translations into sensorial experiences developed by established and emerging artists. Using electronic and digital art, bioart, sculpture and installations, sound and performance, the authors are removing boundaries between natural and artificial, real and imaginary, science and culture. The book aims to hybridize art projects and transdisciplinary approaches to a contemporary art practice by developing a new understanding of media and an innovative approach to materials in the Anthropocene.”* [9]

**Fig. 4:**
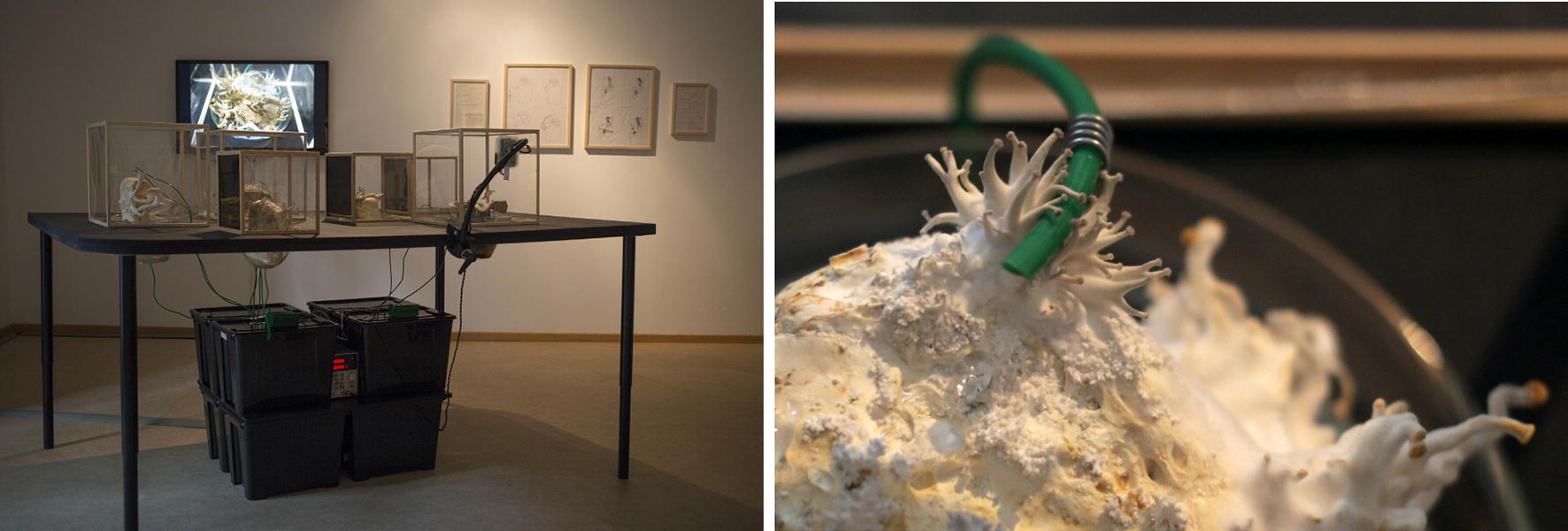
Theresa Schubert: Growing Geometries—Tattooing Mushrooms, exhibition view at Art Laboratory Berlin, 2015

One of Schubert’s artistic long-term studies is titled *Growing Geometries*—*Tattooing Mushrooms* (Fig. 4), which started as part of her Ph.D. research on agency in bio media art at Bauhaus University Weimar in 2015. Reflecting human and nonhuman relationships Schubert investigates “with a focus on methods of generating images by nature. The deeply anthropocentric gesture of tattooing puts the fungi closer to mankind and helps to translate a growth process into an aesthetic experience” [10]. Schubert applies geometric shapes on living fungi with a tattoo machine with coloured ink. The geometric figures on the mushroom’s growing cup constantly alter in time—a wonderful visualized proof of the concept of growth. The complex installation encompasses living fungi, Raspberry Pi, camera, screen, video, tattoo machine, drawings and photographs (Schubert has tested the project with various mushrooms, e.g. *Pleurotus ostreatus, Agaricus bisporus*, and *Agrocybe aegerita*; *Lentinula edodes, Macrolepiota procera*).

“This artistic project investigates the morphology of fungi and evolution of geometrical shapes on living and growing membranes and ultimately how images can be generated by natural processes” [10]. In a fascinating way this work connects both the philosophical reflection on time scale, biological time, and various sign systems. While the tattooed squares repeatedly became circles in the fungal caps, we can encounter here a critical take on modernist Bauhaus-traditions whose predominant reference system to geometrical forms contradict(ed) in so many ways bio(morphic) phenomena.

“The cultivation of mycelium and the choice of fungi as material underlie my interest in rhizomatic structures as a biological and sociological phenomenon” remarks Schubert, “not least influenced by Deleuze/Guattari’s application of the rhizome as a metaphor for a poststructuralist model of knowledge organization and distributed organization without hierarchies.”^8^ Reflecting this piece under the paradigm of human–nonhuman hierarchy, there is a phenomenal detail in the project’s outcome. As Schubert apparently was confronted repeatedly with the question of collaboration, she added a new layer to this project this year—as if to do justice to the mushroom as equal co-agent: After choosing one of her drawings of the growing mushrooms of the same project she had it tattooed on her own body (on her back): “I feel that now this project is completed for me because after tattooing the mushrooms, I am now closing the loop back to myself, making my skin available and also going through the painful process of this aesthetic inscription method” [10].

**Fig. 5:**
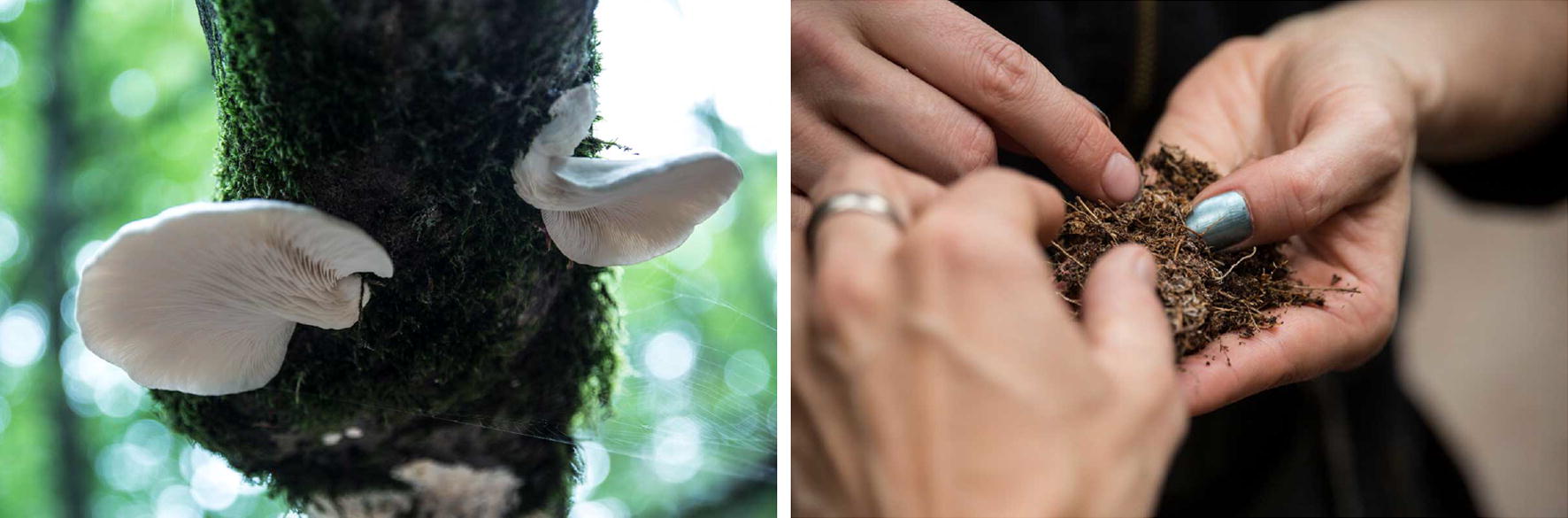
Theresa Schubert: *Forestal Psyche*, Performative Walk through the Briesetal Forest, Brandenburg, August 2017, Photos: Tim Deussen

In context of Schubert’s various formats and the methods of her biomedia practice with and about mushrooms, she also had undertaken several performative methods to encounter human–nonhuman entanglements with mushrooms in situ—together with the public: In summer 2017 Schubert realized her project *The Forestal Psyche* including a public walk in the forests of Brandenburg, outside Berlin (Fig. 5). The public was invited to universally interact with the natural forest habitat, that Schubert has used in her art. By introducing the group into the world of lichen, slime moulds and fungi found on the forest walk, the artist took the role of both performer and (inter)mediator. The collected samples were further analysed under field microscopes and discussed within the group.

“I wanted to see how imagination and biotechnology would work together in an artistic workshop”, the artist reflects in an essay retrospectively on her forestal intervention. “Is it possible to demystify hard science by interpreting it creatively? Following Barad’s reading of performance as a scientific practice I understood myself as part of the investigation in an interconnected world” [10].

**Fig. 6:**
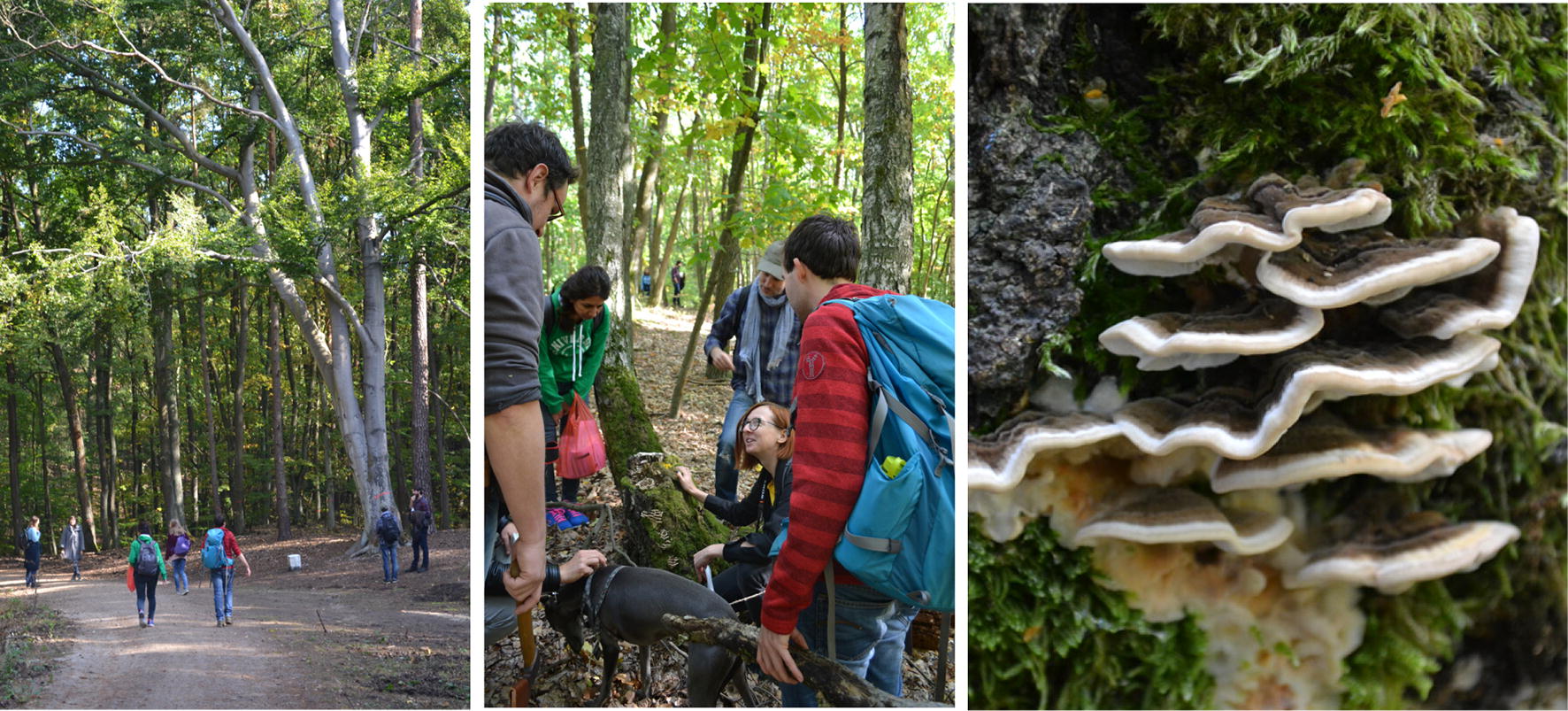
Theresa Schubert: Walk & Talk/Mind the Fungi, October 2018, Tegel Forest, Photos: Art Laboratory Berlin

Once part of Art Laboratory Berlin’s series “Nonhuman Agents”—Schubert continued and enhanced her fascinating forestal walk in a different, but not completely new context: in the current art science research project *Mind the Fungi*, a collaboration between the Institute of Biotechnology TU Berlin and Art Laboratory Berlin (2018–2020) [11]. Focussing on local tree mushrooms as sustainable material for the future this interdisciplinary research project invited Schubert as artist-in-resident to research, explore, experiment together. One of her contributions were several *Walk & Talks* in October and November 2018 in Berlin and Brandenburg forests with a broad public (Fig. 6). What philosopher and physicist Barad had once formulated in reference to material engagement and scientific research: “A performative understanding of scientific practices, for example, takes account of the fact that knowing does not come from standing at a distance and representing but rather from a direct *material engagement with the world*”.

Schubert has offered diverse and meaningful perspectives on fungi and the forest and cultural historical approaches. Her open formats, like talks and performances in situ, play a vital role in the context of art science research, especially connected to a *citizen scientist* component. As Schubert is very experienced in this she is the one who bridges the gap between public, scientists and knowledge—both informatively and aesthetically, logistically and formally. It was amazing to witness during the last Walk & Talks the collaboration between artist and the scientists, as their collective knowledge, approaches and methods led to a universal unique experience for the public. In a more and more complex world, this seems to be undoubtedly one of the most sustainable ways of knowledge collection and dissemination.

## Discussion

The series of “Nonhuman Subjectivities” (2016/17) as well as “Nonhuman Agents” (2017/18) allowed me to not only curate and discuss new artistic approaches connected to nonhuman agency and sentience. The series also gave me space to theoretically and philosophically explore new works in Hybrid Arts that relate to the idea of human–nonhuman relationships and works with biomaterial *as such*. When living matter becomes a medium, not only for human art production, but for an attempt for cross species ‘intra-action’, what are the implications of this new paradigm of Hybrid Arts?

Working with Saša Spačal and Tarsh Bates in the context of the series of “Nonhuman Subjectivities” (2016/17) as well as “Nonhuman Agents” (2017/18) allowed us to see the manifold complexities of artworks produce in collaboration, not only between artist and scientist, but between the human and nonhuman. *Myconnect* and *The Surface dynamics of adhesion* offer the viewer/participant/recipient an opportunity to experience or contemplate symbiosis. In the former work the experience is explicitly non-linguistic, in the latter is introduced through a subtle ‘Trompe-l’œil’ in which the viewers only slowly discovers that the artist has literally offered her blood as sustenance for the yeast. Theresa Schubert meanwhile sees the nonhuman as co-creator through interventions, both into the fungal morphology and into our own cultural notions of what we call ‘nature’ and how these are currently being eroded by the ‘Anthropocene’.

## Conclusions

As Biology has challenged our notions of what is human, art practice that engages the life sciences has developed new forms and practices. No longer ‘representational’, this art is performative, interactive and multi-disciplinary. Living organisms become medium for an experimental art that not only actively engages science, but seeks to bridge the human and nonhuman. Saša Spačal’s work *Myconnect* creates a functional symbiosis between the human participant and mycelia. Tarsh Bates’s *Surface dynamics of adhesion* involves creating a biotechnological home for a yeast which is both human holobiont and pathogen, within an art installation. Theresa Schubert’s work is a collaboration with the fungi, though an intervention with its morphological growth. We can also consider her approach as an exploration of the forest as a space formerly known as ‘nature’, in an age when humans influence the whole planet. And yet humans are just discovering that we are ourselves intimately interconnected with the world around us on the most intimate microbial and metabolic levels.

## Data Availability

Not applicable.

## Footnotes

1 On the basis of exhibitions, talks, seminars, workshops and a 3-day international interdisciplinary conference entitled “Non-Human Agents in Art, Culture and Theory” in November 2017, a wide discursive field of topics, theses, formats, projects and in particular questions in an open working mode that has been and will be negotiated with a broad public. In 2017, the concept continued under the title “Nonhuman Agents”. About the artists and the concept, see here: http://artlaboratory-berlin.org/html/de-Programm-2016.htm (10.6.2019).

2 “Mycelium’s oscillations of electrical resistance than generate temporal offset of the incoming heartbeat, that is transferred back to the human body via sound, light and haptic sensory impulses.” https://www.agapea.si/en/projects/myconnect (10.6.2019).

3 http://www.agapea.si/en/projects/myconnect (10.6.2019).

4 Many thanks to Lizzy Sawyer for this valuable information. As an ALB team member she was present during the whole exhibition time of “Nonhuman Networks”, where the work *Myconnect* was shown (30 September–26 November 2017).

5 This work was part of the group exhibition “The Other Selves. On the Phenomenon of the Microbiome” at Art Laboratory Berlin (27 February–30 April 2016), http://artlaboratory-berlin.org/html/eng-exh-39.htm (10.6.2019).

6 Although she had to write a 70-page safety document, and had the institution of SymbioticA and the Anatomy Dept. of UWA on her side.

7 In 2018, Bates exhibited this piece again in the Hygiene Museum in Dresden, when *Candida parapsylosis* had been reclassified as level 2. She therefore chose a third level 1 *Candida*.

8 During the 1970s French philosopher Gilles Deleuze and French psychoanalyst Felix Guattari developed the term “rhizome” and “rhizomatic” to stress non-hierarchic systems of thought, working with planar and trans-species connection. They used “rhizomatic” in opposition to arborescent (for hierarchic, tree-like), which they would use for hierarchic structures of knowledge. See Gilles Deleuze/Félix Guattari: *Capitalisme et Schizophrénie*, Paris 1972 (vol 1), 1980 (vol 2); Gilles Deleuze/Félix Guattari: *Mille Plateaux*, Paris 1980.
